# Ventricular Tachycardia due to an Overdose of Tibetan Drugs: A Case Report of Aconitine Poisoning

**DOI:** 10.1002/ccr3.70538

**Published:** 2025-05-28

**Authors:** Panjing Liu, Mingyuan Niu, Xiaobin Zhang, Hui Han

**Affiliations:** ^1^ Department of Cardiovascular Medicine People's Hospital of Shigatse City Shigatse P.R. China; ^2^ Department of Emergency People's Hospital of Shigatse City Shigatse P.R. China; ^3^ Department of Emergency Renji Hospital, Shanghai Jiao Tong University School of Medicine Shanghai P.R. China; ^4^ Department of Cardiovascular Medicine Ruijin Hospital, Shanghai Jiao Tong University School of Medicine Shanghai P.R. China

**Keywords:** aconitine poisoning, case report, Tibetan drug, ventricular tachycardia

## Abstract

Tibetan medicine enjoys a high recognition among Tibetans. Many Tibetan hospitals and medicine shops are dedicated to related research and prescribe Tibetan drugs to patients. Overdose intake of aconite‐containing Tibetan drugs might cause severe poisoning manifesting malignant ventricular arrhythmias. Supportive measurements, including external chest compression, electric cardioversion, fluid resuscitation, and intravenous administration of amiodarone/lidocaine, aim at restoring sinus rhythm, serving as “time‐buying” mechanisms to allow the body to naturally excrete the toxic alkaloids.

**Summary**

- *Aconitum pendulum* is a representative of the commonly used poisonous plants in Tibetan medicine.
- The rapid onset of life‐threatening heart rhythm disorders might occur following an overdose of aconite‐containing Tibetan drugs.
- These arrhythmias could be managed conservatively allowing the toxin to flush itself out of the system.

## 1 Introduction

Based on the theory of “Four Tantras” (*rgyud bzhi* in the Tibetan language), Tibetan ethnomedicine has an important status both in traditional Chinese medicine and in the world medicine system [1]. *Aconitum pendulum* is a representative of the commonly used poisonous plants in Tibetan medicine. Among the diterpenoid alkaloids isolated from *Aconitum pendulum*, aconitine has attracted the most attention for its high toxicity and wide range of bioactivities [1]. Aconite‐containing Tibetan drugs, mostly pills in dosage form, are easily obtained from local Tibetan medicine shops and are very commonly used among middle‐aged and elderly Tibetan farmers and herdsmen. Pharmacologically, *Aconitum pendulum* can be developed as analgesic and anti‐rheumatic agents due to its antinociceptive and anti‐inflammatory effects [2, 3]. However, the margin between therapeutic and toxic doses is narrow (< 20% difference between median effective dose and median lethal dose) [4]. The potential severity of aconite poisoning is related to the rapid onset of life‐threatening heart rhythm disorders [5].

This report details a case of a 53‐year‐old male who exhibited refractory ventricular tachycardia and cardiac shock after overdosing on a Tibetan drug (Qiong'a, or Wupeng Wan) containing *Aconitum pendulum, Myrobalan, Rhizoma acori calami, Radix aucklandiae*, and *Musk*. His clinical condition improved following frequent electric cardioversions combined with the use of antiarrhythmic agents including amiodarone and lidocaine.

## 2 Case History/Examination

A 53‐year‐old Tibetan farmer (height: 176 cm, weight: 88 kg) was admitted to our hospital with general weakness, dizziness, nausea and vomiting, and numbing limbs after taking an overdose of Wupeng Wan (containing about 150 mg *Aconitum pendulum* extracts, four times of the maximum recommended dose) 1 h before admission. By taking excessive medication, he intended to quickly control his discomfort caused by “cold” History was negative for headache, chest pain, shortness of breath, or abdominal pain. He was previously healthy without hypertension, diabetes mellitus, heart disease, chronic obstructive pulmonary disease, and cerebrovascular disease. Serum electrolytes (K^+^, Na^+^, Cl^−^, and Ca^2+^) were normal. Blood cell count and other chemistry values were all included in Table 1.

**TABLE 1:** Blood cell count and other chemistry values.

Variables	Values	Variables	Values
Leucocyte, ×10^9^	11.78	Plasma glucose, mmol/L	7.28
Neutrophil, ×10^9^	5.38	K, mmol/L	4.00
Lymphocyte, ×10^9^	5.34	Na, mmol/L	139.8
Eosinophil, ×10^9^	0.12	Cl, mmol/L	104.3
Basophil, ×10^9^	0.05	Ca, mmol/L	2.54
Erythrocyte, ×10^12^	6.55	PT, s	10.40
Hemoglobin, g/L	197	PT‐INR	0.90
Platelet, ×10^9^	256	APTT, s	27.2
ALT, U/L	59	D‐dimer, mg/L	0.20
AST, U/L	54	Myoglobin, ng/mL	167.6
Albumin, g/L	49	CK‐MB, ng/mL	2.4
TBil, μmol/L	21.7	hsTnI, ng/mL	0.0096
SCr, μmol/L	107	hsCRP, mg/L	< 0.5
UA, μmol/L	601	PCT, ng/mL	0.075

Abbreviations: ALT, alanine aminotransferase; APTT, activated partial thromboplastin time; AST, Aspartate aminotransferase; CK, creatine kinase; hsCRP, hypersensitive C‐reactive protein; hsTnI, high‐sensitivity troponin I; PCT, procalcitonin; PT, prothrombin time; PT‐INR, prothrombin time‐international normalized ratio; SCr, serum creatinine; TBil, total bilirubin; UA, uremic acid.

## 3 Differencial Diagnosis, Investigations, and Treatment

**FIGURE 1:**
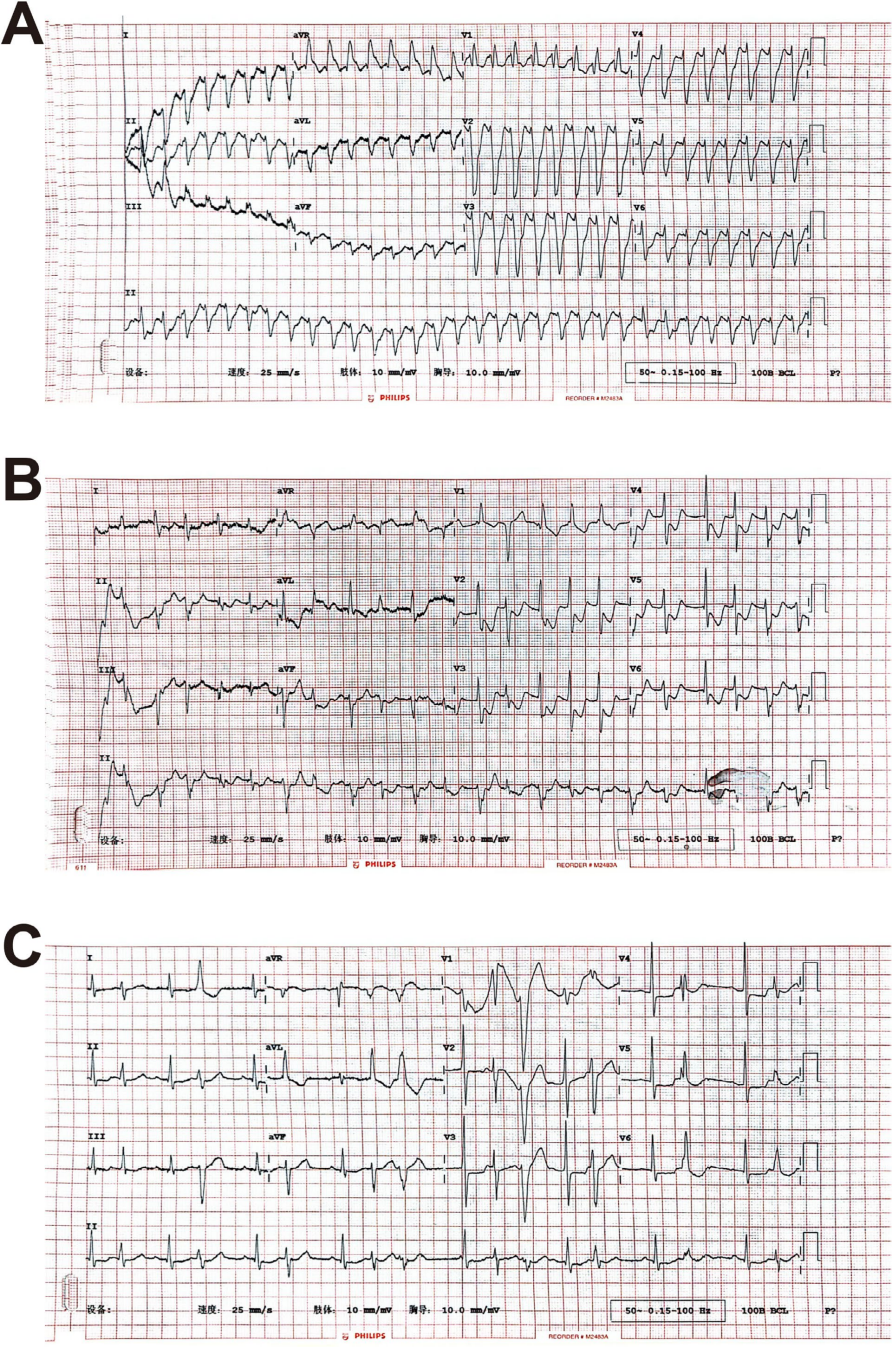
EKG changes during emergency treatment. (A) Persistent ventricular tachycardia. (B) Prolonged QTc interval. (C) Frequent ventricular premature contractions. QTc interval, corrected QT interval.

**FIGURE 2:**
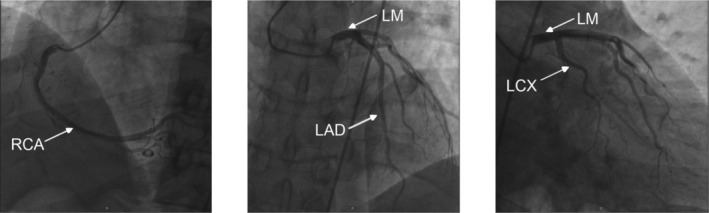
Normal coronary angiography results on Day 1. LAD, left anterior descending; LCX, left circumflex; LM, left main; RCA, right coronary artery.

Upon receiving gastric lavage in the emergency room, the patient suddenly experienced a disturbance of consciousness due to persistent ventricular tachycardia (Figure 1A). Though external chest compression and several attempts at electric cardioversion (200 J, non‐synchronized) were performed immediately, sinus rhythm could not be maintained even after the injection of amiodarone (150 mg loading dose over 10 min, followed by 60 mg/h via the jugular vein). The prolonged QTc interval limited the continued use of amiodarone (Figure 1B). Thus, we replaced amiodarone with lidocaine (100 mg loading dose over 10 min, followed by a continuous infusion at 0.03 mg/kg/min). Fluid resuscitation was carried out throughout the treatment to control hypotension. After 60 min of emergency treatment, the patient's consciousness returned and sinus rhythm maintenance was finally achieved, although there were still frequent ventricular premature contractions (Figure 1C). Subsequently, coronary angiography was performed, which yielded normal results (Figure 2).

## 4 Conclusion and Results

**FIGURE 3:**
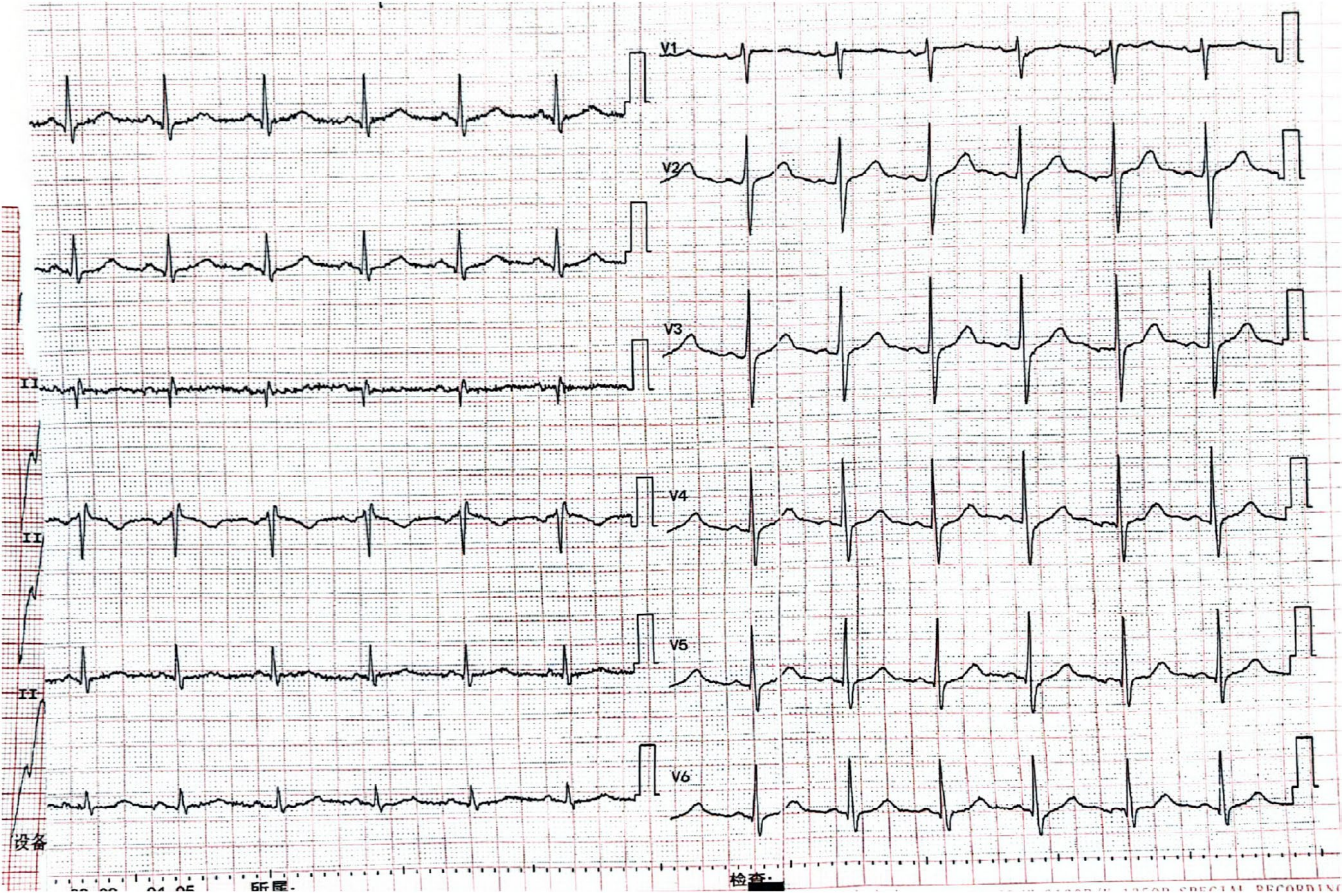
Normal EKG results on Day 3.

The patient was discharged from the cardiac care unit (CCU) on Day 3 with a normal EKG (Figure 3) and an excellent left ventricular performance (LVEF 65%, Table 2). The patient did not come back to our hospital after his discharge.

**TABLE 2:** Characteristics of echocardiography.

Variables	Values	Variables	Values
EF, %	65	Aortic root diameter, mm	31
FS, %	36	PA diameter, mm	23
LVEDD, mm	47	RVEDD, mm	32
LA dimension, mm	35	RA dimension, mm	37
LVPWd thickness, mm	9	RVPW, mm	3
IVSd thickness, mm	9		

Abbreviations: EF, ejection fraction; FS, fractional shortening; IVSd, interventricular septum diastole; LA, left atrium; LVEDD, left ventricular end‐diastolic diameter; LVPWd, left ventricular posterior wall diastole; PA, pulmonary artery; RA, right atrium; RVEDD, right ventricular end‐diastolic diameter; RVPW, right ventricular posterior wall.

## 5 Discussion

We reported a case of aconite poisoning due to an overdose intake of Tibetan drugs. Cardiac dysrhythmias, such as sustained ventricular tachycardia, paroxysmal atrial fibrillation, frequent pre‐ventricular contractions, and intraventricular blocks, were observed during emergency treatment. The EKG abnormalities were resolved after electric cardioversions, fluid resuscitation, and intravenous administration of amiodarone/lidocaine.

Aconite poisoning is familiar to clinicians practicing in Tibet, since extracts from the aconite plant are frequently used in Tibetan medicines. Aconite alkaloids are highly lipophilic and can be rapidly absorbed through the upper gastrointestinal tract [6]. Commonly, toxicity manifests within 30–90 min after ingestion and may persist for 24–48 h [4]. In this case, gastrolavage was performed 1 h after drug misuse, but this attempt did not prevent the occurrence of subsequent malignant arrhythmias. Our patient developed a combination of cardiovascular, neurologic, and gastrointestinal signs and symptoms.

Aconite toxicity is mainly induced by aconitine, a 19‐carbon diterpenoid ring‐structured alkaloid that tends to bind to excitable tissues, including myocardium, nerves, and muscle [7, 8]. The voltage‐dependent sodium channels are the principal molecular targets of aconitine. The binding of aconitine promotes the prolongation of the open state of voltage‐dependent sodium channels, resulting in sustained membrane depolarization [5, 9, 10]. In the context of cardiac function, prolonging sodium current influx impedes cardiac repolarization and initially increases cellular excitability, promoting premature excitation. Death usually results from resistant malignant ventricular arrhythmias, refractory shock, or respiratory paralysis occurring within the initial 24 h.

Unfortunately, no specific antidote was found up to now. The treatment of aconitine poisoning is mainly supportive, including gastric lavage, hemodynamic support (fluid resuscitation, cardiopulmonary resuscitation [CPR] and cardiopulmonary bypass) and agents to reduce vagal tone [11]. The management of malignant ventricular arrhythmias remains controversial. Reasonable and feasible options include sodium channel blockers (procainamide, lidocaine, mexiletine, flecainide, etc.), amiodarone, electric cardioversion, magnesium sulfate, and charcoal hemoperfusion, while none of these attempts is uniformly effective [6, 12, 13]. In order to confront hemodynamic instability, we immediately implemented prolonged CPR and fluid resuscitation through the jugular vein. Electric cardioversion, amiodarone, and lidocaine were successively employed to successfully restore sinus rhythm in the present case. The whole process lasted about 60 min. In the real world, amiodarone and lidocaine are still the most efficient drugs for malignant ventricular arrhythmias in emergency treatment. In our case, amiodarone failed to maintain sinus rhythm. Considering the prolonged QTc interval, probably the effect of amiodarone might increase the incidence of torsade de pointes, we replaced amiodarone with lidocaine and achieved favorable results. If these measures failed to maintain hemodynamic stability, we might consider ventilator‐assisted ventilation and cardiopulmonary bypass. Three days after this emergency treatment, the patient enjoyed a normal cardiac rhythm and function, suggesting that aconitine might have little direct lethal damage to cardiomyocytes.

In conclusion, overdose intake of aconite‐containing Tibetan drugs (analgesic and anti‐inflammatory agents) might cause severe aconitine poisoning. Although aconitine poisoning does occur, cases presenting with malignant arrhythmia are not common. Early identification, diagnosis, and treatment are essential for potential patients. For those suffering malignant ventricular arrhythmias, supportive measurements and therapies aiming at restoring sinus rhythm serve as “time‐buying” mechanisms to allow the body to naturally excrete the toxic alkaloids, despite no specific antidote having been discovered. As for preventive measures, imposing restrictions and warning signs on the use of aconite‐containing Tibetan drugs might be an efficient strategy. Our successful experience provided important preliminary data on treating aconitine poisoning in real‐world clinical practice.

## Author Contributions

**Panjing Liu:** data curation, writing – original draft. **Mingyuan Niu:** data curation, writing – review and editing. **Xiaobin Zhang:** writing – review and editing. **Hui Han:** investigation, supervision, validation, writing – review and editing.

## Consent

Written informed consent was obtained from the patient to publish this report in accordance with the journal's patient consent policy.

## Conflicts of Interest

The authors declare no conflicts of interest.

## Data Availability

The data that support the findings of this study are available on request from the corresponding author. The data are not publicly available due to privacy or ethical restrictions.
